# Complete Genome Sequence of *Mycoplasma arginini* Strain HAZ 145_1 from Bovine Mastitic Milk in Japan

**DOI:** 10.1128/genomeA.00265-15

**Published:** 2015-04-16

**Authors:** Eiji Hata

**Affiliations:** Dairy Hygiene Research Division, National Institute of Animal Health (NIAH), National Agriculture and Food Research Organization (NARO), Sapporo, Hokkaido, Japan

## Abstract

*Mycoplasma arginini* is a species sometimes isolated from bovine specimens, mastitic milk, etc. Its pathogenicity against cows, however, is unspecific, unlike other bovine mycoplasmas. Its whole-genome sequence is needed to comprehend its real image. We present here the 678,592-bp complete genome sequence of *M. arginini* strain HAZ 145_1.

## GENOME ANNOUNCEMENT

*Mycoplasma arginini* is a mycoplasmal species that has been isolated from various mammalian host and cell cultures, and also from bovine specimens, mastitic milk, etc. (1, 2). *M. arginini* may become a precipitating factor for bovine mastitis by other bacteria, but its pathogenicity against cows is unspecific (3). Although a draft genome sequence of *M. arginini* has already been reported (4), the complete genome sequence has not. We present here the whole-genome sequence of strain HAZ 145_1, which was isolated in 2008 from bovine mastitic milk in Japan.

Total genomic DNA was prepared from *M. arginini* strain HAZ 145_1 and subjected to 454 Titanium sequencing at the Hokkaido System Science Co., Ltd., Sapporo, Japan. The resulting DNA fragments (reads) were assembled *de novo* using GS De Novo Assembler software v2.7 (Roche), yielding 41 contigs with 100.3 × coverage. An analysis of the contig ends, together with PCR amplification and amplicon cloning, showed that the 678,592-bp genome had a closed-ring structure. After the initial automated annotation performed using the Microbial Genome Annotation Pipeline v2.18 at the DNA Data Bank of Japan (http://migap.ddbj.nig.ac.jp/mgap/jsp/index.jsp) (5–7), manual curation was performed, followed by verification of potential pseudogenes by PCR and Sanger sequencing. As a result, we confirmed 518 open reading frames, 10 pseudogenes, 33 tRNAs, and two sets of each rRNA (5S rRNA, 16S rRNA, and 23S rRNA) in this genome sequence. Moreover, the G+C content was 26.38%.

*M. arginini* is an arginine-metabolizing bovine mycoplasma, like *Mycoplasma alkalescens* and *Mycoplasma canadense*. Most genes containing rRNA in *M. arginini* strain HAZ 145_1 exhibited high similarity to the amino acid sequences of the genes encoded by *M. canadense* strain HAZ360_1 (8).

The enzymes involved in the arginine hydrolysis pathway and acetate kinase play an important role in energy generation in arginine-metabolizing mycoplasma, and genes coding these enzymes were found in this genome sequence (9, 10). On the other hand, genes of proteins involved in the synthesis of capsular polysaccharides and the production of active oxygen-containing molecules, suggested to be important mycoplasmal etiologic agents (i.e., UTP-glucose-1-phosphate-uridyltransferase or glycerol-3-phosphate oxidase, etc.), were not confirmed (11, 12).

The hypothetical proteins MARG 145_0741 may be involved in the antigenic variation shift in surface proteins that plays a role in the adaptation to new surroundings and in host defense mechanisms (8, 12, 13). A part of the amino acid sequences of this gene showed certain similarity to the hypothetical protein MCAN 360_0280, MCAN 360_0281, and MCAN 360_0504 in *M. canadense* strain HAZ360_1 (8). Moreover, the discriminative homopolymeric tract of contiguous thymines and adenines [poly(TA)] is located upstream of repetitive regions in this gene (8, 12, 13). The gene contains homologous regions, which consist of periodic 82-amino-acid sequences.

The genomic sequence of *M. arginini* will provide a foundation for the investigation of this species in the future. Ultimately, it is hoped that this study will contribute to the reduction of mycoplasmal bovine diseases.

### Nucleotide sequence accession number. 

The whole-genome sequence has been registered at DDBJ/EMBL/GenBank under the accession no. AP014657.
